# Lambert & Co.

**Published:** 1884-01-20

**Authors:** 


*Lambert & Co.*—We have tested the preparation, LISTERINE, in practice, and find it to be a neat, convenient and excellent antiseptic preparation. Attention is also invited to a medicine prepared by the same excellent house styled Lithiated Hydrangia. See advertisement.

